# Dietary Phenolic Compounds Interfere with the Fate of Hydrogen Peroxide in Human Adipose Tissue but Do Not Directly Inhibit Primary Amine Oxidase Activity

**DOI:** 10.1155/2016/2427618

**Published:** 2016-01-05

**Authors:** Christian Carpéné, Mounia Hasnaoui, Balázs Balogh, Peter Matyus, Alfredo Fernández-Quintela, Víctor Rodríguez, Josep Mercader, Maria P. Portillo

**Affiliations:** ^1^Institut des Maladies Métaboliques et Cardiovasculaires, Institut National de la Santé et de la Recherche Médicale (INSERM U1048), I2MC, 31432 Toulouse Cedex 4, France; ^2^Université Paul Sabatier, I2MC-UPS, CHU Rangueil, 31432 Toulouse Cedex 4, France; ^3^Department of Organic Chemistry, Semmelweiss University, Hőgyes Endre Utca 7, Budapest 1095, Hungary; ^4^Nutrition and Obesity Group, Department of Nutrition and Food Science, University of the Basque Country (UPV/EHU) and Lucio Lascaray Centre, 01006 Vitoria, Spain; ^5^CIBERobn Physiopathology of Obesity and Nutrition, Institute of Health Carlos III (ISCIII), 28029 Madrid, Spain

## Abstract

Resveratrol has been reported to inhibit monoamine oxidases (MAO). Many substrates or inhibitors of neuronal MAO interact also with other amine oxidases (AO) in peripheral organs, such as semicarbazide-sensitive AO (SSAO), known as primary amine oxidase, absent in neurones, but abundant in adipocytes. We asked whether phenolic compounds (resveratrol, pterostilbene, quercetin, and caffeic acid) behave as MAO and SSAO inhibitors. AO activity was determined in human adipose tissue. Computational docking and glucose uptake assays were performed in 3D models of human AO proteins and in adipocytes, respectively. Phenolic compounds fully inhibited the fluorescent detection of H_2_O_2_ generated during MAO and SSAO activation by tyramine and benzylamine. They also quenched H_2_O_2_-induced fluorescence in absence of biological material and were unable to abolish the oxidation of radiolabelled tyramine and benzylamine. Thus, phenolic compounds hampered H_2_O_2_ detection but did not block AO activity. Only resveratrol and quercetin partially impaired MAO-dependent [^14^C]-tyramine oxidation and behaved as MAO inhibitors. Phenolic compounds counteracted the H_2_O_2_-dependent benzylamine-stimulated glucose transport. This indicates that various phenolic compounds block downstream effects of H_2_O_2_ produced by biogenic or exogenous amine oxidation without directly inhibiting AO. Phenolic compounds remain of interest regarding their capacity to limit oxidative stress rather than inhibiting AO.

## 1. Introduction

Resveratrol is a well-known nonenzymatic antioxidant molecule and it has been reported to exert neuroprotective actions for more than a decade [1]. Recently,* trans*-resveratrol and* cis*-resveratrol have been described as inhibitors of recombinant human monoamine oxidases, MAO-A and MAO-B, the two forms of amine oxidases (AO) involved in neurotransmitter metabolism and in the scavenging of endogenous or exogenous amines [2]. Later on,* trans*-resveratrol has been evidenced to dose-dependently inhibit MAO-A in mouse brain in a manner that may participate to its antidepressant effects, while it was less active towards MAO-B [3], a finding which is in agreement with the pioneering observations of Zhou et al. [4]. Such resveratrol/MAO interaction has attracted great interest since MAOs can be considered as targets of therapeutic approaches for neurodegenerative and psychiatric disorders. However, none of these former studies were performed with the native forms of the human MAO flavoproteins naturally expressed in brain or peripheral tissues. In addition, at least a couple of questions were elicited by this incompletely deciphered interaction between resveratrol and MAO. (1) Does resveratrol interact only with brain MAO or with other members of the AO family? (2) Can phenolic antioxidants other than resveratrol interact with AOs? This context prompted us to further study the interplay between phenolic compounds and AOs.

The AO family encompasses FAD-dependent enzymes (essentially MAO and polyamine oxidase) and copper-containing amine oxidases, having for predominant members the products of the genes AOC1, 2, and 3, the latter being named semicarbazide-sensitive amine oxidase (SSAO) or primary amine oxidase and also currently known as vascular adhesion protein-1 (SSAO/VAP-1) [5, 6]. Many molecules that interact with MAO, even in an inhibitory manner, may also interact with other AOs [7]. To give only a mere example, it can be mentioned that phenelzine, a well-recognized MAO inhibitor used as an antidepressant drug, also inhibits SSAO/VAP-1 [8, 9] and possesses neuroprotective properties [10, 11]. While the different members of the AO family are encoded by distinct genes and exert various biological functions, they have only few specific substrates and inhibitors, since numerous endogenous or exogenous amines exhibit poor selectivity towards the diverse AOs (for an exhaustive review, see the book: [12]). Irrespective of its nature, any enzyme of the AO family oxidizing an amine in the presence of water and dioxygen is releasing the corresponding aldehyde, ammonia, and hydrogen peroxide [13]. Even though being not a radical, the latter end-product, H_2_O_2_, is a member of the reactive oxygen species (ROS) that readily participate in oxidative stress. Thus, AOs can be included in the cellular ROS-generating systems [13]. We therefore investigated the putative interplay between phenolic compounds (natural ingredients endowed with antioxidant properties) and AO enzymes, supposed to act in an opposite manner on oxidative stress.

Indeed, considering that various so-called MAO inhibitors can also interact with other AOs, we asked whether resveratrol, known to exert antioxidant activities, known to activate sirtuins [14, 15], and recently described to inhibit MAO [2, 16], was able to interact with SSAO, too. Another line of observations also prompted us to perform such verification. It is now established that phenolic compounds exhibit anti-inflammatory effects [17, 18]. Taking into account the fact that many inhibitors of SSAO/VAP-1 (engineered antibodies or soluble small molecules) are also endowed with potent anti-inflammatory actions in various experimental models [19–21], it was of relevant interest to determine whether resveratrol might also inhibit SSAO/VAP-1 and whether such putative inhibition was contributing to its anti-inflammatory actions. All these considerations led us to test whether several phenolic compounds could inhibit human MAO and SSAO/VAP-1 and whether such inhibition may account for a portion of their multiple effects.

Therefore, the objective of our work was focused on testing the capacity, if any, of selected phenolic compounds on the activity of these enzymes under their native form in man, since interspecies differences have been reported regarding substrate and inhibitor selectivity for MAOs and SSAO [22, 23]. Among the stilbenes, we compared* trans*-resveratrol (the most abundant form in foods and beverages) to pterostilbene, one of its hydroxylated derivatives, since both of them have been reported to repress adipogenesis in fat cells [24–26] (see below). Caffeic acid was chosen as representative of the phenolic acids since it has also been demonstrated to be inhibitory on adipogenesis [27]. We also included quercetin in our comparative study, since it is quantitatively the most important phenolic compound present in foodstuffs (as indicated by the phenolic compounds database: http://www.phenol-explorer.eu/ [28]). The following results obtained on human adipose tissue are not only relevant for obesity research but also valuable for oxidative stress aspects because AOs are ROS-generating enzymes and because phenolic compounds, though being among the most important low molecular weight molecules absorbed from foods capable of eliciting nonenzymatic antioxidant actions in consumers, are scarcely described to impair MAO [2–4].

## 2. Materials and Methods

### 2.1. Subjects and Adipose Tissue Sampling

Samples of subcutaneous abdominal adipose tissue were obtained from a total of 39 overweight women undergoing reconstructive surgery at Rangueil Hospital, Toulouse, France (mean age: 39 ± 2 years, range: 18–66, and mean body mass index: 25.36 ± 0.62 kg/m^2^). The removed pieces of human adipose tissue (hAT), considered as surgical waste, were transferred in less than one hour to the laboratory, under the agreement of INSERM guidelines and ethic committee. These hAT pieces were frozen at −80°C and dispatched for further analyses. Moreover, part of the adipose samples was immediately subjected to liberase digestion at 37°C to obtain freshly isolated adipose cells for glucose transport assays as detailed below.

### 2.2. Amine Oxidase Activity Measurement by Fluorometric Determination of Hydrogen Peroxide

Oxidase activity was measured using the fluorescent probe Amplex Red (10-acetyl-3,7-dihydrophenoxazine) designed for the detection of hydrogen peroxide in biological milieu. Assays were performed accordingly to our previous descriptions [29, 30], which corresponded to slight modifications from the adaptation of such fluorometric method for MAO activity determination [31]. Briefly, hydrogen peroxide release was quantified owing to a chromogenic mixture containing 40 *μ*M Amplex Red and 4 U/mL horseradish peroxidase and to the parallel use of hydrogen peroxide standard solutions ranging from 0.05 to 5 *μ*M (final concentrations) [32]. Thawed hAT samples were homogenized in 200 mM phosphate buffer (pH 7.4) during 30 sec with a homogenizer Tissue Master-125 (Omni International, Kennesaw, GA, USA) just prior to the determination of their amine oxidase activity, as previously reported [32], except that no antiprotease cocktail was added. Homogenates were distributed in 96-well dark microplates (at 50 ± 5 *μ*g protein/well) and incubated at 37°C in the dark for at least 30 min in 200 *μ*L final volume after a 30 min preincubation without (control) or with 1 mM pargyline or semicarbazide to inhibit MAO or SSAO activity, respectively, or even with both reference inhibitors to abolish all AO activity. All phenolic compounds tested for their inhibitory properties were also added during this preincubation step. Tyramine or benzylamine was added to the medium in order to obtain 1 mM concentration and to serve as substrates for AO activities, as already stated [29]. The DMSO vehicle used for phenolic compounds solubilization was present at the final higher concentration of 1% w/v and inhibited amine-induced signal by less than 5%.

Spontaneous hydrogen peroxide formation by homogenates of thawed material was also quantified without any addition of exogenous amine or inhibitor and defined as basal release. The influence of chemical compounds or biochemical agents on the fluorescence readouts generated by the hydrogen peroxide standard curve was also performed without any presence of hAT in a set of experiments called “without biological material.” In all cases, fluorescence data (ex/em: 540/590 nm) were collected on a Fluoroskan Ascent plate reader (ThermoLabsystems, Finland).

### 2.3. Radiochemical Assay of Amine Oxidase Activity

Samples of adipose tissue were homogenized as described above. 50 *μ*L of crude homogenates were then incubated for 30 min at 37°C in 200 *μ*L of 200 mM phosphate buffer in the presence of radiolabelled amine after 30 min preincubation without or with inhibitor(s). MAO-dependent oxidation was defined as sensitive to inhibition by 1 mM pargyline, whereas SSAO-dependent oxidation was abolished by 1 mM semicarbazide. Assays were started by addition of the labelled substrate and stopped by adding 50 *μ*L of 4 M HCl. Reaction products of amine oxidation were extracted by 1 mL of organic solvent (toluene/ethyl acetate, 1/1 v/v), according to Tipton's method [23]. Then, 0.7 mL aliquots of the organic phase were counted for radioactivity. Maximal velocity and optimal conditions for determination of amine oxidase activities have been already detailed for human adipocytes, cultured preadipose cells, or hAT homogenates [33–35]. Thus, inhibition studies were performed only under conditions reaching maximal oxidation velocity, that is, corresponding to the use of tyramine or benzylamine isotopic dilutions giving 1 mM final concentration and reaching approximately 640000 dpm/50 *μ*L for [^14^C]-tyramine (provided either by PerkinElmer, Evry, France, or by Sigma-Aldrich) or 200000 dpm/50 *μ*L for [^14^C]-benzylamine (NEC 835050UC purchased from PerkinElmer). The radioactivity extracted in the organic phase at time 0 (*t*0) represented less than 0.5% of the total radioactivity/tube of each labelled amine and was subtracted to all counts with tyramine and benzylamine oxidation without inhibitor averaging 14160 ± 5620 dpm (*n* = 16) and 15800 ± 1770 dpm (*n* = 12), respectively. These absolute values, which varied substantially from one individual to another, were set as 100% reference in each subject for the calculation of percentages of inhibition.

### 2.4. Hexose Uptake in Adipocytes

To determine glucose transport activity, hAT was grossly minced and digested at 37°C under shaking in 20 mL of Krebs-Ringer medium containing 0.015 mg/mL liberase (type TM, Roche Diagnostics), 15 mM sodium bicarbonate, 10 mM HEPES, and 3.5% bovine serum albumin. Buoyant adipocytes were separated by filtration through nylon screen and carefully washed in the same medium at pH 7.4 without liberase to obtain adipocyte suspensions as already described [36]. Freshly isolated adipocytes were incubated for 45 min with the tested agents just before [^3^H]-2-deoxy-glucose uptake assays (PerkinElmer) performed in 10 min at 37°C in plasticware as already described [37].

### 2.5. Chemicals

Tyramine hydrochloride, benzylamine hydrochloride, amine oxidase inhibitors, quercetin,* trans*-resveratrol, and other reagents were obtained from Sigma-Aldrich (Saint-Quentin-Fallavier, France), except otherwise specified.

### 2.6. Computational Studies of Molecular Docking in the Active Site of Amine Oxidases

The structure of* trans*-resveratrol (https://pubchem.ncbi.nlm.nih.gov/compound/445154) was used for computational docking in the structure of MAO-A cocrystallized with harmine at 2.2 Å resolution (http://www.rcsb.org/pdb/explore.do?structureId=2Z5X), MAO-B cocrystallized with coumarin-analogue (https://pubchem.ncbi.nlm.nih.gov/compound/11616886) at 1.7 Å resolution (http://www.rcsb.org/pdb/explore/explore.do?pdbId=2V61), and SSAO/VAP-1 cocrystallized with a pyridazinone analogue at 2.8 Å resolution (http://www.rcsb.org/pdb/explore/explore.do?pdbId=4BTW). Flexible ligand approach with standard precision (SP) was performed with Schrödinger's software package (release 2015-2), using Glide software version 6.7 (http://www.schrodinger.com/Glide/). The phenolic compounds were treated as noncovalent ligands to dock them into the substrate pocket: next to FAD in MAO-A (about 4 Å distance) and MAO-B (about 5.5 Å distance) and next to the topaquinone residue (about 8.5 Å distance) in SSAO/VAP-1. Docking boxes were positioned on the cocrystallized ligands, the size of the boxes was 20 Å in every direction, and no constrains were used.

### 2.7. Statistical Analysis

Results are given as means ± standard error of the means (SEM). Statistical significance was assessed by use of Student's *t*-test. Significance level was set at *P* < 0.05. IC_50_ values were calculated by nonlinear regression using GraphPad Prism (CA, USA).

## 3. Results

### 3.1. ROS Release by Human Subcutaneous Adipose Depots in Response to Amines

**Figure 1:**
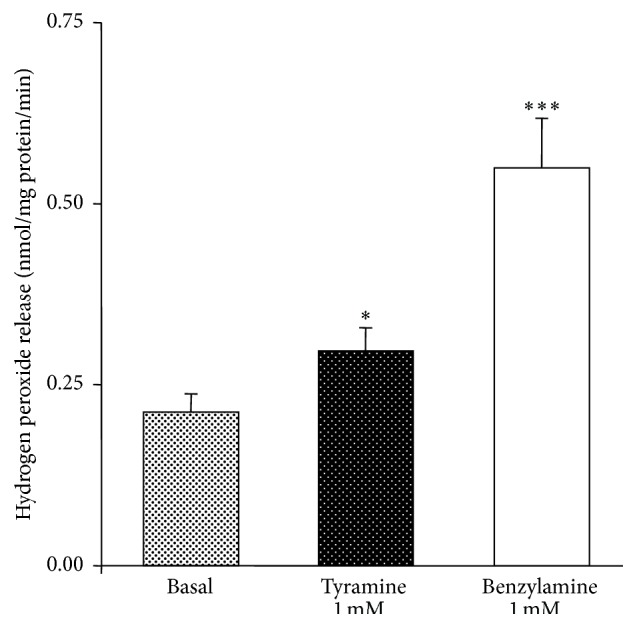
Hydrogen peroxide release by human subcutaneous adipose tissue: influence of amine substrates. Amplex Red-based fluorescence detection of hydrogen peroxide was performed in homogenates during 30 min incubation without any added amine (basal) or with 1 mM of the indicated amine. Results are expressed as velocity of hydrogen peroxide production per mg of protein per minute. Mean ± SEM of 22 homogenates. Different from basal at ^*∗*^ *P* < 0.05 and ^*∗∗∗*^ *P* < 0.001.

Spontaneous and amine-stimulated hydrogen peroxide production by hAT preparations was measured on 30 min incubation (Figure 1). When prolonging incubation conditions it was observed that such ROS release was linear with time for at least one hour (not shown). Benzylamine, and tyramine to a lesser extent, significantly increased the amount of detected hydrogen peroxide. Since the chromogenic mixture was already present in the incubation medium at time 0 when the amines were added and since the net fluorescent intensity was calculated as the difference between* t*30 and* t*0 signal, the observed increase in amount of hydrogen peroxide corresponded to a real time-dependent H_2_O_2_ production by hAT preparations and its subsequent release into the medium. Such widely recognized method of AO activity determination consisted in monitoring the release of only one of the oxidative deamination end-products, hydrogen peroxide. On the basis of the stoichiometry of the oxidative deamination, it was supposed that one molecule of H_2_O_2_ corresponded to one molecule of amine oxidized by AO. In the absence of amine, there was a spontaneous hydrogen peroxide release: this basal activity represented approximately one-third of the maximal response to benzylamine.

The amine-induced hydrogen peroxide production was then used to test a putative interaction of phenolic compounds with human MAO and SSAO.

### 3.2. Interactions between Phenolic Compounds and MAO- or SSAO-Induced Hydrogen Peroxide Production

**Figure 2:**
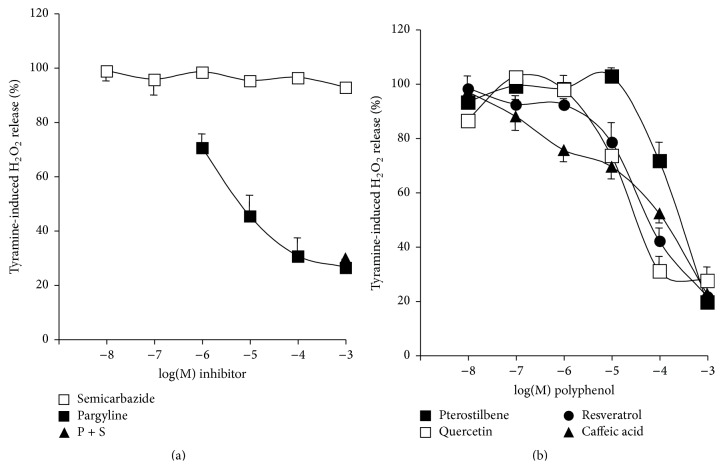
Inhibition of tyramine-induced hydrogen peroxide production in human subcutaneous adipose tissue homogenates by reference MAO and SSAO inhibitors and by phenolic compounds. Fluorescence readouts after 30 min incubation were substracted from corresponding values at* t*0 and net increase was set at 100% for 1 mM tyramine without any inhibitor. (a) The MAO (pargyline, dark squares) or SSAO inhibitor (semicarbazide, open squares) was present during the 15 min preincubation and the 30 min incubation at 0.001–1 mM or at 0.01 *μ*M–1 mM, respectively. The addition of pargyline 1 mM + semicarbazide 1 mM (P + S, black triangle) was used to abolish all amine oxidase activity. Mean ± SEM of 6 to 22 homogenates. When invisible, SEM bars lie within the symbols. (b) The phenolic compounds were tested at the indicated final doses during preincubation and incubation: resveratrol (dark circles), pterostilbene (dark squares), caffeic acid (black triangles), or quercetin (open squares). Mean ± SEM of 7 to 10 homogenates.

It was tested whether the response to tyramine was sensitive to reference inhibitors: pargyline (MAO-selective) and semicarbazide (SSAO-selective). The former inhibited dose-dependently tyramine action, while the latter was totally inefficient (Figure 2(a)). Of note, the combination of both inhibitors did not inhibit more than pargyline alone, leaving unaltered approximately 30% of the production found in the presence of tyramine. This confirmed that, in human fat stores, tyramine was mainly oxidized by MAO. When phenolic compounds were studied in identical conditions, all the four tested molecules (see Figure 7 for chemical structures) reached at 1 mM the same maximal inhibition of tyramine-induced H_2_O_2_ release (Figure 2(b)). At the 1 *μ*M dose, all were almost unable to modify the response to 1 mM tyramine, as it was the case for the classical AO inhibitors. The rank order of affinity was: quercetin > resveratrol > caffeic acid > pterostilbene, with respective IC_50_ values being 30, 62, 100, and 107 *μ*M. From these observations, it could be deduced that resveratrol and other phenolic compounds were able to limit the tyramine-induced H_2_O_2_ release, that is, to inhibit MAO activity, thus confirming previous reports on resveratrol and expanding to other phenolic compounds the capacity to inhibit MAO.

**Figure 3:**
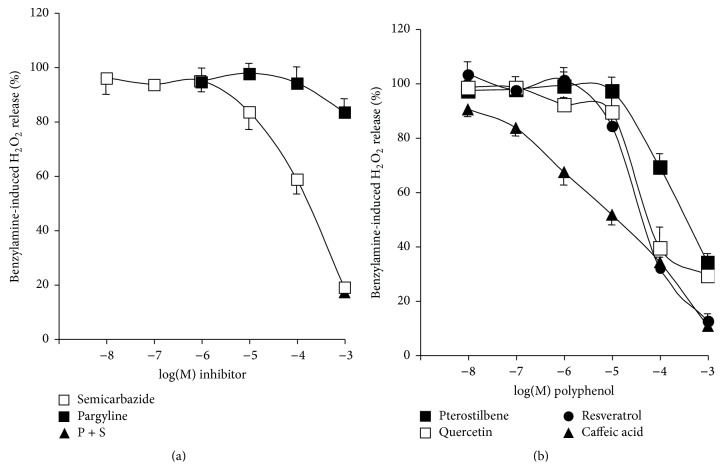
Inhibition of benzylamine-induced hydrogen peroxide release in human subcutaneous adipose tissue by reference MAO and SSAO inhibitors and by phenolic compounds. Fluorescence readouts after 30 min incubation were substracted from corresponding values at* t*0 and net increase was set at 100% for 1 mM benzylamine alone. (a) Pargyline (MAO, dark squares) or semicarbazide (SSAO inhibitor, open squares) was present at indicated final concentrations, while their combination was tested at 1 mM (P + S, black triangle). Mean ± SEM of at least 6 homogenates. (b) The phenolic compounds were tested in parallel at the indicated final concentrations. Means ± SEM of 8 to 12 homogenates.

Figure 3(a) shows that benzylamine-induced hydrogen peroxide release was inhibited by 1 mM semicarbazide while being resistant to pargyline. Again, the combination of pargyline and semicarbazide did not inhibit further than the SSAO inhibitor semicarbazide alone, leaving unaltered 20% proportion of the signal. These data confirmed that benzylamine is predominantly oxidized by SSAO in hAT, as previously reported [34].

Phenolic compounds dose-dependently inhibited benzylamine-induced H_2_O_2_ release (Figure 3(b)), indicating that they apparently behave as SSAO inhibitors. In this case, the IC_50_ values (*μ*M) for caffeic acid, resveratrol, quercetin, and pterostilbene were 36, 45, 69, and 136, respectively. However, resveratrol and caffeic acid abolished benzylamine-induced signal at 1 mM, almost to a deeper extent than the reference inhibitor semicarbazide, while quercetin and pterostilbene impaired partially the benzylamine-induced signal.

This dissimilarity prompted us to further verify whether the phenolic compounds were impairing the generation of hydrogen peroxide or whether they blunted its detection by the chromogenic mixture used for fluorometric detection.

### 3.3. Phenolic Compounds Reduce Hydrogen Peroxide Induced Fluorescence of Amplex Red-Based Chromogenic Mixture

**Figure 4:**
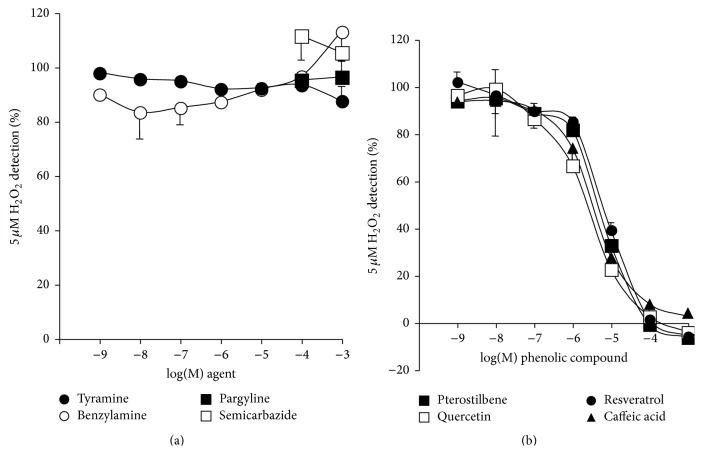
Influence of amine oxidase substrates or inhibitors and phenolic compounds on fluorescent-based detection of hydrogen peroxide. The chromogenic mixture used for Amplex Red-based fluorometric detection of hydrogen peroxide was incubated with 5 *μ*M H_2_O_2_ alone (control set at 100%) or in the presence of the indicated final concentrations of tested chemicals. (a) Lack of influence of MAO and SSAO substrates or inhibitors. (b) Dose-dependent abolishment by phenolic compounds of the fluorescent signal elicited by 5 *μ*M hydrogen peroxide. Means ± SEM of 7 measurements without any human material.

At this stage, it appeared necessary to determine whether the detection of hydrogen peroxide could be directly influenced by phenolic compounds irrespective of its source: production by AOs, generation by other enzymatic systems, or even exogenous addition by the experimenter. Without any human biological material in the assays, the classical substrates and inhibitors of MAO and SSAO did not alter the fluorescence found in the presence of 5 *μ*M hydrogen peroxide (signal set at 100%) even when present at 0.1–1 mM (Figure 4). DMSO was also inactive at the dose used as a vehicle for polyphenol solutions (1%, not shown). On the opposite side, a strong interaction with fluorescence readouts was found with the four tested phenolic compounds in the range of 1 *μ*M–1 mM. Figure 4 clearly shows that all the tested phenolic compounds dose-dependently lowered with almost the same potency the fluorescent signal elicited by 5 *μ*M hydrogen peroxide. Corresponding EC_50_ values were as follows in *μ*M: quercetin 2.0, caffeic acid 3.5, resveratrol 5.0, and pterostilbene 5.2. With 1 mM phenolic compounds, there were even unexplained low readouts that were lower than the blank made with phosphate buffer and chromogenic mixture only, leading to percentages lower than 0%. All this indicated that the phenolic compounds prevented detection of hydrogen peroxide under optimal conditions since they hindered signal generation, likely by preventing the interaction between H_2_O_2_ and chromogenic mixture components (Amplex Red, peroxidase, or resorufin). Such interaction likely relies on the antioxidant properties of the tested phenolic compounds and deserves further investigation. However, our objective was to assess whether MAO and/or SSAO activities were really altered by resveratrol and related molecules. We therefore used another method to measure more directly amine oxidation, based on the use of radiolabelled substrates.

### 3.4. Influence of Phenolic Compounds on [^14^C]-Tyramine Oxidation

**Figure 5:**
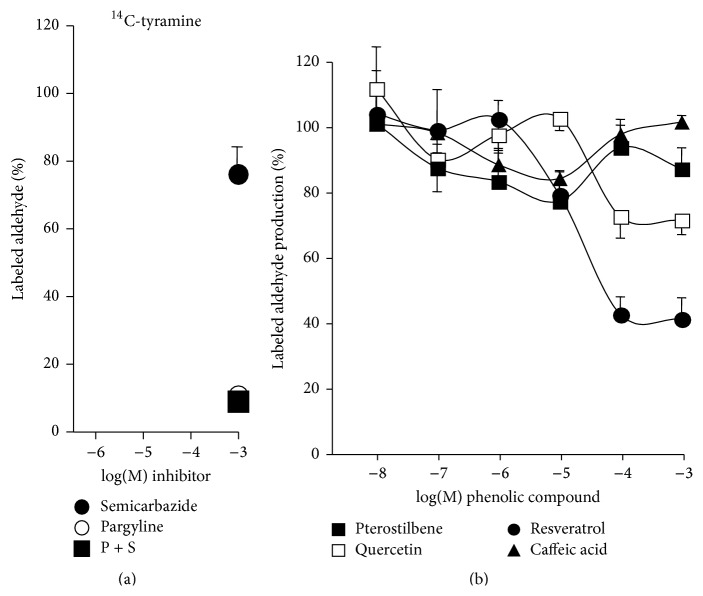
Influence of phenolic compounds and MAO inhibitors or SSAO inhibitors on tyramine oxidation by hAT. The production of radiolabelled aldehydes resulting from the 30 min incubation of fat tissue homogenates with 1 mM isotopic dilution of [^14^C]-tyramine was set at 100% in the absence of added inhibitor, while the background radioactivity detected at* t*0 was set at 0%. (a) Inhibition by pargyline only of MAO-dependent tyramine oxidation. (b) Influence of phenolic compounds on tyramine oxidation. Each point is the mean ± SEM of 8 (inhibitors) to 12 (phenolic compounds) homogenates.

Figure 5 shows that pargyline inhibited the MAO-dependent [^14^C]-tyramine oxidation by hAT. As expected, and accordingly with the above-used method, semicarbazide was virtually unable to abolish this activity. Surprisingly, phenolic compounds did not inhibit MAO activity totally. Only resveratrol and quercetin exhibited partial MAO inhibitor properties, since, at 0.1–1 mM, they inhibited 25 to 60% of the activity. Contrasting with the fluorescent method, pterostilbene and caffeic acid did not abolish enzyme activity when taking into account the production of labelled oxidation products of [^14^C]-tyramine (Figure 5).

### 3.5. Interaction of Phenolic Compounds with SSAO-Dependent Oxidation of [^14^C]-Benzylamine

**Figure 6:**
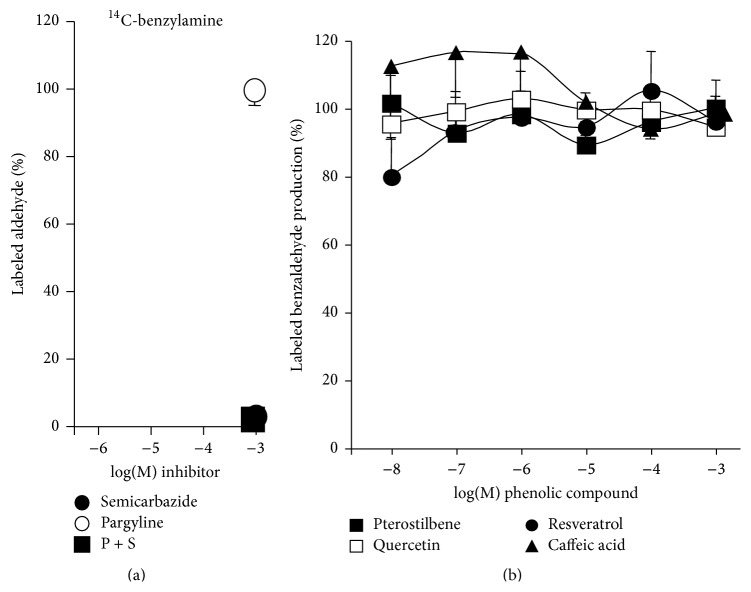
Influence of phenolic compounds and MAO inhibitors or SSAO inhibitors on benzylamine oxidation by hAT. (a) Thirty-minute oxidation of 1 mM [^14^C]-benzylamine by homogenates generated amounts of radiolabelled benzaldehyde that were separated by extraction as described in Materials and Methods and set at 100% signal (control), while 0% corresponded to the extractible radioactivity at* t*0. The reference MAO inhibitors (pargyline) or SSAO (semicarbazide) inhibitors were present, separately or in combination at 1 mM, 15 min before the addition of the radiolabelled substrate. (b) Indicated doses of resveratrol (dark circles), pterostilbene (dark squares), caffeic acid (black triangles), or quercetin (open squares) and pargyline (dark circles) were tested on the oxidation of 1 mM [^14^C]-benzylamine in the same conditions as for reference inhibitors. Each point is the mean ± SEM of 8 homogenates.

As expected, benzylamine oxidation by hAT was sensitive to semicarbazide. The addition of both pargyline and semicarbazide could not inhibit further than semicarbazide alone, which eradicated the generation of labelled benzaldehyde. The SSAO-mediated [^14^C]-benzylamine oxidation remained unchanged with low or high doses of phenolic compounds (Figure 6). Taken as a whole, these data indicated that SSAO/VAP-1 was the unique catalyst participating in benzylamine oxidation and that pargyline as well as polyphenols could not prevent this type of amine metabolism.

Therefore, phenolic compounds, which inhibited benzylamine-induced signal in fluorometry, were likely preventing H_2_O_2_ from reacting with chromogenic mixture (as this was the case without biological material) and lowered fluorescent readouts rather than really inhibiting benzylamine oxidation and subsequent H_2_O_2_ release. In addition, it could be mentioned briefly here that SSAO-mediated oxidation of [^14^C]-benzylamine was not altered by Amplex Red, peroxidase, or DMSO at the doses used for the fluorometric method or by catalase or even by H_2_O_2_ (not shown).

**Figure 7:**
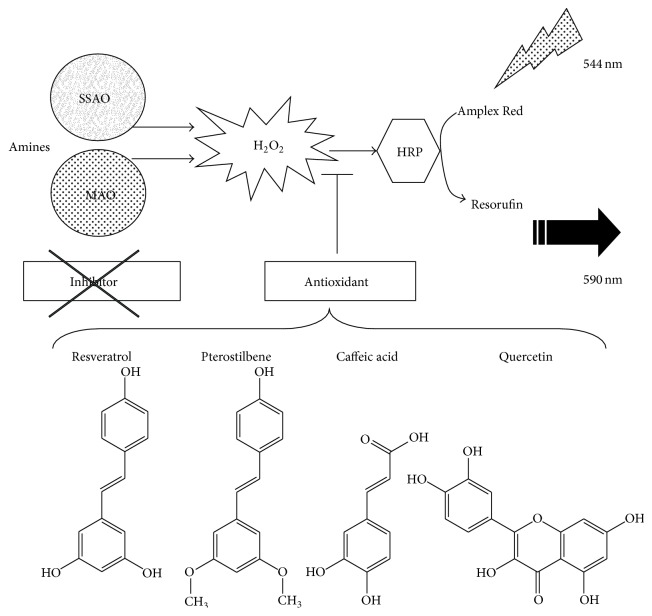
Chemical structures and proposed mechanism of action of phenolic compounds in the Amplex Red-based fluorometric determination of amine oxidase activity in tissue preparations. The tested phenolic compounds with given structures did not behave as full inhibitors of MAO and SSAO present fat tissue, since only resveratrol partially inhibited [^14^C]-tyramine oxidation by human subcutaneous adipose tissue. However, all of them inhibited hydrogen peroxide detection by preventing the oxygen species from reacting with horseradish peroxidase (HRP) and hampering the generation of the oxidized Amplex Red, that is, the fluorescent probe, resorufin.

To summarize, resveratrol, quercetin, pterostilbene, and caffeic acid actually impaired the detection of the produced hydrogen peroxide without preventing benzylamine degradation and behaved as MAO inhibitors and SSAO inhibitors only in appearance, as schematized in Figure 7. Only resveratrol and quercetin shared a limited capacity to alter partially the MAO activity found in hAT homogenates, at doses between 0.1 and 1 mM, that is, at concentrations that can be qualified as supranutritional.

### 3.6. Computational Docking in the Active Site of Amine Oxidases

**Figure 8:**
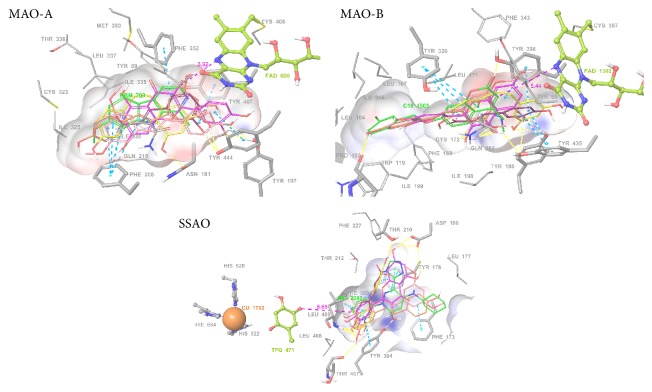
Docking poses of resveratrol, pterostilbene, quercetin, and caffeic acid in the active sites of human MAO-A, MAO-B, and SSAO. For all enzymes, amino acid residues surrounding the catalytic site are shown and numbered in grey, while for MAO-A and MAO-B, FAD cofactor is shown in green yellow. For SSAO, topaquinone and copper are shown in green and orange. Green: cocrystallized inhibitor (see Section 2). Yellow:* cis*-resveratrol. Orange:* trans*-resveratrol. Pink: pterostilbene. Purple: quercetin. Maroon: caffeic acid. *π*-*π* interactions are marked with light blue dotted lines, while H-bond interactions are marked with yellow dotted lines.

Calculations were performed to simulate the docking of the four tested phenolic compounds into human MAO-A, MAO-B, and SSAO. All these small soluble molecules were able to approach very nearly the catalytic sites of the oxidases (Figure 8).

In the case of MAO-A, all the tested agents could dock in a close vicinity to the FAD. Possible aromatic *π*-*π* interactions were observed with Phe208, Phe352, Tyr407, and Tyr444. Some H-bonds were also formed between the hydroxyl groups of the ligands and polar amino acids Asn181, Tyr197, and Tyr444. Docking scores calculated by glide varied between −8.300 and −5.682 with the standard precision method (SP) and between −11.568 and −5.563 with the extra precision level (XP). The predicted activity order was as follows: quercetin >* cis*-resveratrol >* trans*-resveratrol > pterostilbene > caffeic acid, with both SP and XP.

In the case of MAO-B, our computational studies allowed adding resveratrol to the list of chemical compounds that are not amines but which noncovalently bind to MAO within a wide range of affinity: diphenylbutane, farnesol, coumarin…, and so on. The formation of *π*-*π* interactions was indicated with the aromatic rings of Tyr326, Tyr398, and Tyr435; H-bonds were found with Cys 172, Tyr188, and Tyr435. The backbone oxygen atom of Pro102 seemed to be an H-bond acceptor for several ligands. Scores were slightly higher than those with MAO-A, since SP scores ranged from −9.312 to −6.849 and XP scores ranged from −11.020 to −6.997.

Regarding SSAO, both* cis*-resveratrol and* trans*-resveratrol could be docked into the pocket surrounding the cocrystallized inhibitor (a pyridazinone analogue) though with very small scores, indicating that there is a possible binding position but not ascertaining that resveratrol can behave as a noncovalent inhibitor. Indeed, the docking of the stilbene, as well as that of the other phenolic compounds tested, was established relatively far away from both topaquinone and the copper ion, two mandatory cofactors for the enzyme catalysis. *π*-*π* stacking interactions were formed with the following residues: Phe173, Tyr176, Phe389, and Tyr394; H-bonds were formed with Asp180, Thr210, and Tyr394. Backbone oxygen atom of Thr467 and backbone nitrogen atom of Leu469 could also form additional H-bonds. Docking scores were weaker than those with MAO-A and MAO-B: SP scores varied between −6.857 and −5.156, and XP scores varied between −9.261 and −4.100. These weaker scores of the tested polyphenols were in agreement with their lack of inhibition of [^14^C]-benzylamine oxidation.

Though being not all genuine AO inhibitors, the tested natural phenolic compounds could nevertheless interact with the MAO and SSAO roles since they were able to modify the fate of one of their common end-products of reaction, namely, H_2_O_2_. It was therefore investigated whether the phenolic compounds were modulating the response to benzylamine in human adipose cells.

### 3.7. Phenolic Compounds and Benzylamine-Stimulated Glucose Uptake in Human Adipocytes

**Figure 9:**
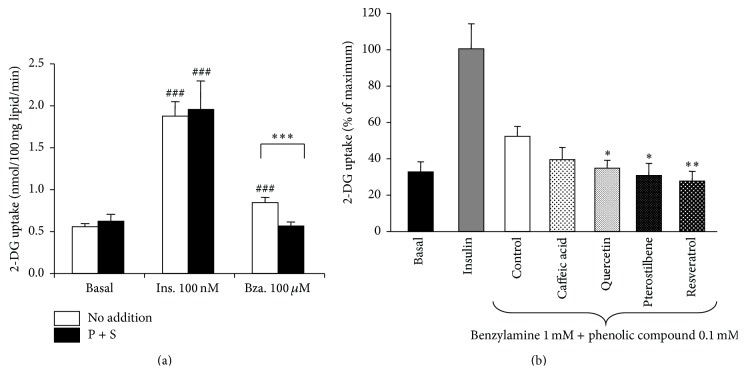
Effects of phenolic compounds, benzylamine, and insulin on glucose transport in human adipocytes. Freshly isolated adipocytes were incubated for 45 min without (basal) or with insulin 100 nM (ins.) or with benzylamine and phenolic compounds at the indicated doses. Then 2-DG uptake assays were performed on 10 min as detailed in Section 2. (a) Blockade of benzylamine insulin-like stimulation of glucose uptake in human adipocytes by amine oxidase inhibitors. Basal, insulin-stimulated, or benzylamine-stimulated 2-DG uptake was tested without (no addition, open columns) or with pargyline + semicarbazide at 1 mM each (P + S, dark columns). Mean ± SEM of 39 cases. Different from basal alone at ^###^ *P* < 0.001. Different from benzylamine alone at ^*∗∗∗*^ *P* < 0.001. (b) Insulin was used as the agent of reference for maximal transport stimulation, set at 100%. Benzylamine was tested at 1 mM alone (control, open column) or in the presence of 100 *μ*M of the indicated phenolic compounds. Mean ± SEM of 7 observations. Different from benzylamine alone at ^*∗*^ *P* < 0.05 and ^*∗∗*^ *P* < 0.01.

It was confirmed that the previously reported insulin-like effect of benzylamine on glucose uptake was readily detectable in human adipocytes from subcutaneous abdominal depots.  Figure 9 shows that, in human fat cells, 100 nM bovine insulin stimulated basal glucose transport by 3-fold to 4-fold. At 100 *μ*M, benzylamine partially mimicked the action of the pancreatic hormone, since its activation of hexose uptake averaged 25 to 30% of maximal insulin effect. However, this modest insulin-like effect was highly significant and demonstrated to be dependent on amine oxidation. Indeed, semicarbazide, together with pargyline, did not modify basal or insulin-stimulated glucose uptake, while it clearly inhibited benzylamine action (Figure 9).

On the other hand, phenolic compounds were tested on 1 mM benzylamine stimulation of deoxyglucose uptake and the results were expressed as percentage of insulin maximal stimulation (which at 100 nM elicited a 3-fold to 4-fold activation over baseline in a subset of 7 individuals, Figure 9). At 100 *μ*M, resveratrol, pterostilbene, and quercetin counteracted the effect of 1 mM benzylamine. No clear-cut inhibition was found with caffeic acid. Although phenolic compounds did not inhibit SSAO activity in hAT, they were likely altering the fate of the hydrogen peroxide produced during SSAO activation by benzylamine. We propose that this was preventing the interaction of hydrogen peroxide with intracellular components, such as phosphatases, already reported to mediate the actions of SSAO substrates on glucose carrier translocation [38].

Therefore, when directly applied to human adipose cells, phenolic compounds appeared to behave as antioxidants preventing hydrogen peroxide actions. Nevertheless they impaired responses to AO substrates, especially those dependent on hydrogen peroxide, such as SSAO-mediated activation of glucose transport.

## 4. Discussion

The present study of polyphenol properties was performed with samples of human subcutaneous abdominal adipose depots. In the following lines we justify this tissue selection. Brain contains neuronal MAO, but its SSAO expression is limited to vessels and meninges [39], while liver is rich in MAO and also expresses SSAO/VAP-1 [40]. However, these tissues are not readily available from healthy volunteers. On the opposite side, hAT offered valuable advantages: it is considered as a surgical waste when obtained in healthy patients undergoing plastic surgery and is able to oxidize amines much more efficiently than blood [41]. Yet, hAT has been shown to express the mitochondrial MAO, with an approximate ratio of MAO-A to MAO-B of 80/20 [33], and to be one of the richest tissues in SSAO/VAP-1 [34]. In fact, SSAO is present at the cell surface of adipocytes and is even considered as a marker of adipogenesis [35, 42].

Our first set of observations clearly showed that resveratrol and other phenolic compounds hampered the detection of hydrogen peroxide generated during benzylamine oxidation by hAT preparations, naturally rich in SSAO/VAP-1 [34]. These results were obtained by using a method that allows measuring MAO or SSAO/VAP-1 activity, based on a fluorometric detection in which Amplex Red is oxidized to resorufin (the real fluoroprobe) by a peroxidase, in a manner that depends on the hydrogen peroxide generated during amine oxidation [31]. Though such method has been already employed with human fat tissue preparations [30], our test of the capacity of polyphenols to inhibit human MAO and SSAO/VAP-1 was accompanied with a complete check aiming at verifying whether they interfere or not with Amplex Red-based fluorescence assay. Such verification avoided misinterpretations of the observed fluorescence readouts, since numerous natural functional ingredients have led to erroneous signals, owing to uncorrected quenching and autofluorescent or antioxidant properties [43].

Thus, the “older” radiochemical determination of AO activity [23] solved the inconsistencies found with the fluorometric method. By using radiolabelled substrates and measuring after extraction of the neosynthesized labelled aldehydes, our comparative approach was able to definitely state about the direct interaction between the tested polyphenols and human MAO or SSAO/VAP-1. By using this analytical method, phenolic compounds were unable to inhibit the oxidative deamination of radiolabelled [^14^C]-benzylamine, suggesting that they were acting as antioxidants rather than genuine SSAO inhibitors (as illustrated in Figure 7). Resveratrol, pterostilbene, quercetin, and caffeic acid could not clearly block AO activities, whereas they could modify the fate of one of the end-products of amine oxidation, hydrogen peroxide. This resulted in an impairment of the interaction between hydrogen peroxide and the chromogenic mixture in fluorometric assays, which could be misinterpreted as an AO inhibition. However, resveratrol and other phenolic compounds altered the consequences of amine oxidase activities that rely on hydrogen peroxide production in hAT, since they were impairing the insulin-like action of benzylamine on glucose uptake in adipocytes. A blockade of these metabolic actions of SSAO substrates has been already reported for SSAO pharmacological inhibitors and for antioxidants (catalase, N-acetylcysteine, and ascorbic acid) [34] but never for natural phenolic compounds.

Thus, our supposed inhibition of SSAO activity by phenolic compounds was not experimentally supported, though a dose-dependent repression of SSAO/VAP-1 was apparent for all phenolic compounds with the misused Amplex Red-based method. However, several pitfalls and complementary experiments rendered irrelevant the shortcut interpretations of fluorescent assays.

Firstly, the fact that resveratrol and caffeic acid totally inhibited benzylamine-induced hydrogen peroxide formation was a little puzzling since the reference inhibitor semicarbazide itself failed to abolish completely the signal. In fact, when hydrogen peroxide formation was measured in the presence of benzylamine, selective pharmacological inhibition of SSAO leaved unaltered approximately 15–20% of the signal. This component was likely independent from SSAO or MAO activity and was resulting from basal ROS production by hAT preparations, as observed in the absence of added amine. A similar spontaneous generation of hydrogen peroxide has been reported in mouse adipose tissue and considered as a baseline ROS production, in which NADPH-oxidase was partly involved [44]. This production represented a minor component compared to that found in response to 1 mM benzylamine treatment. Its detection was impaired by phenolic compounds as was the detection of hydrogen peroxide alone, added at 5 *μ*M in assays without biological material, or even the detection of H_2_O_2_ produced by AO activation.

Secondly, no inhibition of SSAO/VAP-1 was observed with any dose of the four phenolic compounds tested, even up to 1 mM, when using the radiochemical method. This latter determination was undoubtedly more specific than the H_2_O_2_-based assays since the SSAO-dependent proportion (i.e., sensitive to semicarbazide by definition) represented 100% of the [^14^C]-benzylamine-induced response. Obviously, there was no possible spontaneous release of radiolabelled benzaldehyde by hAT preparations not incubated with [^14^C]-benzylamine.

Importantly, the artefacts detected during the determination of SSAO activity also occurred for MAO activity, since the clear-cut inhibition by phenolic compounds of the hydrogen peroxide signal in response to tyramine was not totally reproduced when MAO activity was assessed via the quantification of the radiolabelled end-product of [^14^C]-tyramine oxidation. Only a partial MAO inhibition was found with resveratrol and quercetin during hAT short-term incubations, which was in agreement with their capacity to dock in computed models of the catalytic site of MAO-A and MAO-B.

Thus, our observations contrast with previous reports indicating that resveratrol is a valuable inhibitor of human MAO [2]. In fact, in their pioneering observations, Yáñez and coworkers reported that clorgyline and selegiline were 1000-fold to 10000-fold more potent than resveratrol in an artificial model consisting in insect cells overexpressing the recombinant forms of human MAO-A or MAO-B. Only the comparison with a rather “historical” MAO-inhibitor, iproniazid, that exhibited very poor affinity for the enzymes when compared with novel generation of MAO blockers permitted consideration of the MAO inhibition by 10–100 *μ*M resveratrol of relevant interest [2]. Since Amplex Red was employed in such studies, one can expect that there was an overestimation of the capacity of resveratrol to repress MAO activity. At the same doses, quercetin has been reported already to inhibit MAO-A in a complete manner, though via a peroxidase-based detection of hydrogen peroxide production, therefore probably with a substantial overestimation of its inhibitory properties [45]. Hence, we propose here that resveratrol and quercetin have to be considered as poorly potent MAO inhibitors since they hamper [^14^C]-tyramine oxidation (mainly catalyzed by MAO-A, predominant in human adipocytes) at doses that are closer to the millimolar than to the micromolar range, that is, levels that cannot be reached by nutritional intake. Indeed, maximal plasma resveratrol levels are estimated to reach 3-4 *μ*M after ingestion of 1 mg/kg, according to the freely available database http://www.phenol-explorer.eu/ [28]. Nevertheless, it is important to take into account the possibility that a full mixture of natural phenolic compounds may be effective at lower doses, due to potential synergistic effects.

Many, if not all, effects of resveratrol ingestion should be considered therefore as mainly independent from MAO inhibition. This is not incompatible with the neuroprotective activity of the stilbene since it has been proposed that neuroprotection is not linked to MAO inhibition, even for various MAO inhibitors [9, 11]. An attention that must be given to resveratrol in this view, that is, relative to the highly selective, reversible inhibitors of recent generation with high affinity for MAO, is that it is so far a natural product found in foods and beverages and not a prescribed drug.

Our measurements with the radiochemical method using [^14^C]-benzylamine clearly indicated that resveratrol did not acutely inhibit human SSAO/VAP-1 while semicarbazide did. Of note, semicarbazide used for reference blockade in our assays is no more the best SSAO inhibitor available to date; SSAO has even been renamed primary amine oxidase to signify that it is better defined by its substrates (endogenous or exogenous) rather than by its inhibitor(s) [46]. Although millimolar dose of the reference inhibitor semicarbazide was needed to totally slow down SSAO activity, its blocking action remained selective and was far from being reproduced with the tested phenolic compounds, at least with the radiochemical method.

Most of our computational analyses of resveratrol docking in the catalytic site of human SSAO/VAP-1 were incoherent with a pharmacological binding of high affinity and revealed that SSAO can be hardly considered as a target for this phenolic compound. Regarding MAO, the computationally obtained results were therefore in agreement with the corresponding experimental values and extended the growing list of natural molecules able to interact with functional site of MAOs.

The demonstration that phenolic compounds hampered the detection of hydrogen peroxide when added directly to the fluorometric assay, while not pargyline or semicarbazide, brought further evidence that it was not the generation of hydrogen peroxide occurring during catalyzed oxidative deamination that was prevented by the phenolic compounds but rather the interaction of the MAO-generated or SSAO-generated hydrogen peroxide with chromogenic mixture. On the opposite side, MAO and SSAO genuine pharmacological inhibitors impaired the ROS generation and not its interaction with Amplex Red/peroxidase. Moreover, it was verified that once hydrogen peroxide has completely reacted with the chromogenic mixture the fluorescence readouts were unaltered by subsequent addition of phenolic compounds to the assays. This indicated that phenolic compounds do not quench fluorescence but rather alter the fate of hydrogen peroxide, preventing its interaction with the chromogenic mixture used in our and other fluorometric assays [2] or hampering its messenger role in fat cells (see below).

Though not preventing [^14^C]-benzylamine oxidation, quercetin, pterostilbene, and resveratrol counteracted the benzylamine activation of glucose transport in human adipocytes, a biological effect previously demonstrated to be dependent on hydrogen peroxide [34]. In other words, the fact that phenolic compounds interfere not only with the fluorometric detection of hydrogen peroxide by the chromogenic mixture but also with other fat cell components might have unexpected consequences for future research. Regarding upcoming determinations of MAO and SSAO activities and their interactions with various “novel” agents supposed to act as antioxidants, the verification of putative interaction of these agents with hydrogen peroxide or chromogenic mixture should carefully accompany future tests. Relative to the effectiveness of ingested nonenzymatic antioxidants, it can be advanced that they probably share with classical AO inhibitors the capacity to alter the effects of elevated doses of dietary amines, as illustrated here by the opposite effects of phenolic compounds and benzylamine on glucose transport in human fat cells.

In this view, it is recognized that resveratrol shares with several SSAO inhibitors a strong antiadipogenic action [47–49]. Nonetheless, the stilbene improves glucose tolerance in rodent model of obesity and diabetes [50, 51], while semicarbazide and related agents reduce body weight gain and adiposity without exhibiting noticeable beneficial effect on glucose homeostasis [52, 53]. Curiously, a few drugs share with resveratrol the properties of being both antidiabetic and MAO inhibitors. This is the case of the PPAR*γ* activators known as glitazones, for which the inhibition of the mitochondrial MAO belongs to the list of their “off-target” actions [54], together with their neuroprotective secondary effects [55]. In this line, glitazones have been recently described as MAO inhibitors devoid of SSAO inhibitory properties [30].

## 5. Conclusion

To summarize, the capacity of resveratrol to inhibit MAO-A activity has been confirmed here on the human form in mature human adipocytes that naturally express mainly MAO-A. Nevertheless, we propose that this inhibition has been probably overestimated in various previous reports. We also observed that resveratrol and quercetin could dock into the active sites of human MAO-A and MAO-B with acceptable scores, a finding confirmed during the completion of our work by a recent report of drug-design strategy showing that derivatives of resveratrol plus coumarin were successfully designed to develop a target scaffold to inhibit MAO in a reversible manner [56]. Our limited comparison with four natural phenolic compounds did not indicate which biomolecule could be more beneficial than resveratrol for limiting oxidative stress in experimental models or useful for supplements to human nutrition targeting brain or peripheral MAOs. Nevertheless, our comparative study clearly indicated that none of the tested phenolic compounds directly inhibited primary amine oxidase, also named SSAO/VAP-1. This property cannot be added to their antioxidant action. However, dietary phenolic compounds can hamper the hydrogen peroxide-dependent consequences of SSAO activation, at least in human fat cells. Therefore the coexistence of dietary amines (which will be oxidized once ingested) or other antioxidant molecules present in foods, beverages, or nutritional supplements should be checked carefully. Finally, in a context in which various selective inhibitors of SSAO/VAP-1 have been patented for their anti-inflammatory properties [57, 58], it can be advanced that phenolic compounds exert anti-inflammatory properties independently from direct SSAO/VAP-1 inhibition.
